# Supplementary material for: Azithromycin alters the microbiome composition, function and resistome in women with Chlamydia trachomatis infections
Source: NPJ Biofilms Microbiomes. 2025 Dec 13;11:235. doi: 10.1038/s41522-025-00858-9 (PMC12738766; doi:10.1038/s41522-025-00858-9)

Supplementary Information

Azithromycin alters the microbiome composition, function and resistome in women with *Chlamydia trachomatis* infections

Sankhya Bommana[a*], Olusola Olagoke[a*], Yi-Juan Hu[b], Ruohong Wang[a], Mike Kama[c], Morgan Dehdashti[a], Reshma Kodimerla[a], Timothy D. Read[d¶], Deborah Dean[a,e,f,g,h¶]

[a]Department of Pediatrics, University of California San Francisco, Oakland, CA, USA
[b]Department of Biostatistics and Bioinformatics, Emory University, Atlanta, GA, USA
[c]Ministry of Health and Medical Services, Suva, Fiji
[d]Department of Medicine, Emory University School of Medicine, Atlanta, Georgia, USA
[e]Department of Medicine, University of California San Francisco, San Francisco, CA, USA
[f]Department of Bioengineering, Joint Graduate Program, University of California San Francisco and University of California Berkeley, San Francisco, CA, USA
[g]Bixby Center for Global Reproductive Health, University of California San Francisco, San Francisco, CA, USA
[h]Benioff Center for Microbiome Medicine, University of California San Francisco, San Francisco, CA, USA

Supplementary Figures

Supplementary Figure S1. Alpha diversity measured by Shannon index and log richness of species for the paired vaginal, endocervical and rectal microbiomes for women without treatment, those who cleared their *C. trachomatis* infection post-treatment, and those who had persistent infection after treatment. *P* values are denoted within each box in the lower righthand corner.

Supplementary Figure S2. Beta diversity measured by Jaccard and Bray-Curtis metric for the paired vaginal, endocervical and rectal microbiomes for women without treatment, those who cleared their *C. trachomatis* infection post-treatment, and those who had persistent infection after treatment. *P* values are denoted within each box in the lower righthand corner.

Supplementary Figure S3. Chord diagram representation of the transition in sub-Community State Types (subCSTs) for the three cohort datasets for endocervical, vaginal and rectal sites. *Transition from a *Lactobacillus*-dominated microbiome (subCST I, II or V) to a microbiome dominated by *L. iners* or diverse anaerobic and facultative bacteria (subCST III or IV) or vice versa (‡).

Supplementary Figure S4. Taxonomic profiles of the vaginal microbiomes, paired with the endocervical and rectal microbiomes (not shown), based on relative abundance at the species at baseline and follow-up for the three study cohort datasets. The participant ID# is shown at the top of the respective box that contains baseline (left) and follow-up (right) bar graphs and the in sub-Community State Types (subCSTs) noted below the bar graphs. The samples exhibiting genito-rectal transmissions are shown in **Figure 6**.

Supplementary Figure S5. Taxonomic profiles of the endocervical microbiomes, paired with the vaginal and rectal microbiomes (not shown), based on relative abundance at the species at baselineand follow-up for the three study cohort datasets. The participant ID# is shown at the top of the respective box that contains baseline (left) and follow-up (right) bar graphs and the in sub-Community State Types (subCSTs) noted below the bar graphs. The samples exhibiting genito-rectal transmissions are shown in **Figure 6**.

Supplementary Figure S6. Taxonomic profiles of the rectal microbiomes, paired with the endocervical and vaginal microbiomes (data not shown), based on relative abundance at the species at baseline and follow-up for the three study cohort datasets. The participant ID# is shown at the top of the respective box that contains baseline (left) and follow-up (right) bar graphs and the in enterotype noted below the bar graphs. The samples exhibiting genito-rectal transmissions are shown in **Figure 6**.

Supplementary Figure S7: Function- and pathway-level community analysis of paired vaginal, endocervical and rectal microbiomes of women who did not have a *C. trachomatis* infection and did not receive treatment. A. The Function-level analysis shows the function that had significantly different relative abundances between baseline and follow-up microbiomes for the rectum. No functions were significantly different for the endocervical or vaginal sites. B. The microbial contribution for each function and the associated pathways are shown for baseline and follow up time points. C. The Pathway-level community analysis shows those pathways for each anatomic site that have a significant difference in relative abundance between the baseline and follow-up microbiomes (P<0.05). Species represented in the graphs are color-coded as per the columns to the right.

Supplementary Figure S8. MAFFT alignment of *C. trachomatis rpl*V genes (encoding L22 protein) extracted from baseline and follow-up samples of the *Ct* persistence cohort and baseline samples of the *Ct* clearance group. *Ct* reference strain D/UW-3/CX was used as the sequence to identify the macrolide conferring resistance mutations in our clinical samples. Regions with nonsynonymous missense mutations are shown. Numbering above the nucleotide sequence indicates nucleotide position and numbering below the amino acid sequence indicates amino acid position. The macrolide binding site is noted in blue below the amino acid sequence. Presence of *rpl*V mutations in *Ct* is indicative of macrolide resistance as reported by Mestrovic et al., 2018 and Benamri et al., 2021.

Supplementary Figure S9. MAFFT alignment of *L. iners erm*B genes extracted from baseline and follow-up samples across all three cohorts, *L. iners* strain C0094A1 and C0210C1 harboring *erm*B and the ErmB protein homolog from *Enterococcus faecium* (available from CARD). Regions with nonsynonymous missense mutations are shown. Numbering above the nucleotide sequence indicates nucleotide position and numbering below the amino acid sequence indicates amino acid position. The *L. iners erm*B genes circulating in our microbiomes were found to be >/= 99.1% identical to the protein homolog model, and the strains C0094A1 and C0210C1 were 99.1% identical. Presence of *erm*B in *L. iners* is indicative of macrolide resistance as reported by the CARD model: https://card.mcmaster.ca/ontology/36514.

Supplementary Figure S10. MAFFT alignment of *G. vaginalis erm*X genes extracted from baseline and follow-up samples across all three cohorts, *G. vaginalis* strain JNFY11 harboring *erm*X and the ErmX protein homolog from plasmid pNG2 (available from CARD). Regions with nonsynonymous missense mutations are shown. Numbering above the nucleotide sequence indicates nucleotide position and numbering below the amino acid sequence indicates amino acid position. The. *G vaginalis erm*X genes circulating in our microbiomes were found to be >/= 93.8% identical to the protein homolog model and the strain JNFY11 was 99.3% identical. Presence of *erm*X in *G. vaginalis* is indicative of macrolide resistance as reported by the CARD model: https://card.mcmaster.ca/ontology/36735. Presence of more than one copy of *erm*X represented as 2 next to a given sample ID is indicative of the presence of a second copy of the *G. vaginalis erm*X gene within a microbiome.

Supplementary Tables

Supplementary Table S1. Metagenomic shotgun sequencing results and quality control statistics.

Supplementary Table S2. Metadata of samples used in this study, including data on age, ethnicity, C. trachomatis, BV, pH, *N. gonorrhoeae*, *M. genitalium* and HPV type.

Supplementary Table S3. Distribution of low risk (lr)HPV (black font) and high risk (hr)HPV (red & orange fonts) types in paired vaginal, endocervical and rectal microbiomes.

Supplementary Table S4. Comparison of subCommunity State Type (CST) and metagenomic (mg)CST in paired vaginal and endocervical microbiomes for the three cohort datasets at baseline.

Supplementary Table S5. Comparison of vaginal and endocervical subCST and mgCST classification systems and associations with BV and *C. trachomatis* (*Ct*).

Supplementary Table S6. subCST associations with BV and *C. trachomatis* infection in the vaginal and endocervical microbiomes.

Supplementary Table S7. Functions and pathways associated with the baseline time point for the *C. trachomatis* persistence cohort microbiomes.

Supplementary Table S8. Functions and pathways associated with the follow up time point for the *C. trachomatis* persistence cohort microbiomes.

Supplementary Table S9. Functions and pathways associated with the baseline time point for the *C. trachomatis* clearance cohort microbiomes.

Supplementary Table S10. Functions and pathways associated with the follow up time point for the *C. trachomatis* clearance cohort microbiomes.

Supplementary Table S11. Functions and pathways associated with the baseline time point for the *C. trachomatis* control cohort microbiomes.

Supplementary Table S12. Functions and pathways associated with the follow up time point for the *C. trachomatis* control cohort microbiomes.

Supplementary Table S13. Function-level community analysis of the microbiomes from the *Ct* clearance cohort.

Supplementary Table S14. Function-level community analysis of the microbiomes from the *Ct* persistence cohort.

Supplementary Table S15. Function-level community analysis of the microbiomes from the no treatment cohort.

Supplementary Table S16. Supplementary Table S16. *G. vaginalis erm*X and *L. iners erm*B genes that confer resistance to macrolide antibiotics, and *L. iners tet*(W) gene conferring tetracycline resistance and mutations in the *C. trachomatis rpl*V gene that may confer macrolide resistance identified among endocervical, vaginal and rectal microbiomes for all cohorts at baseline (blue) and follow-up (red).

Supplementary Figure S1

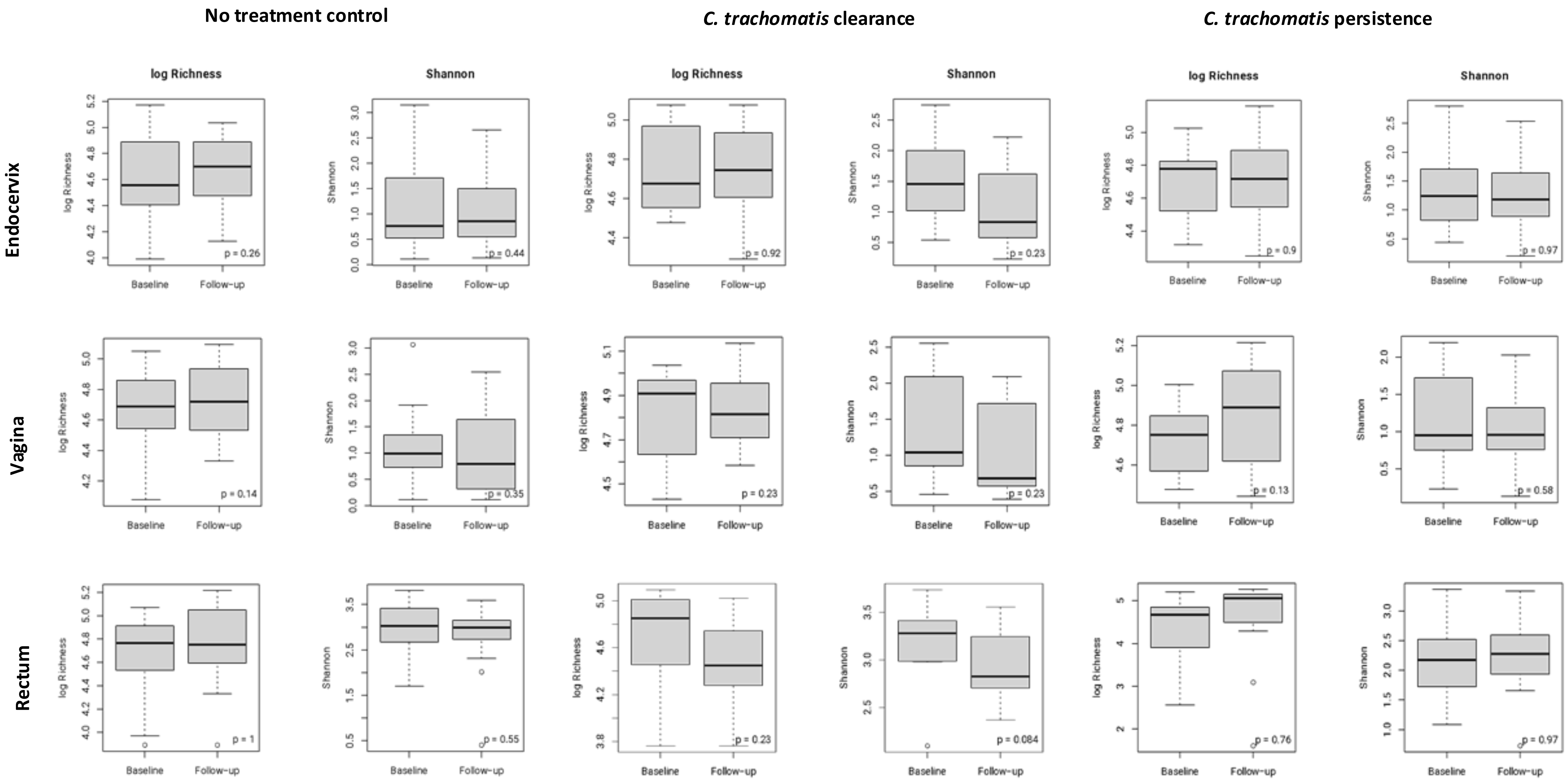

Supplementary Figure S2

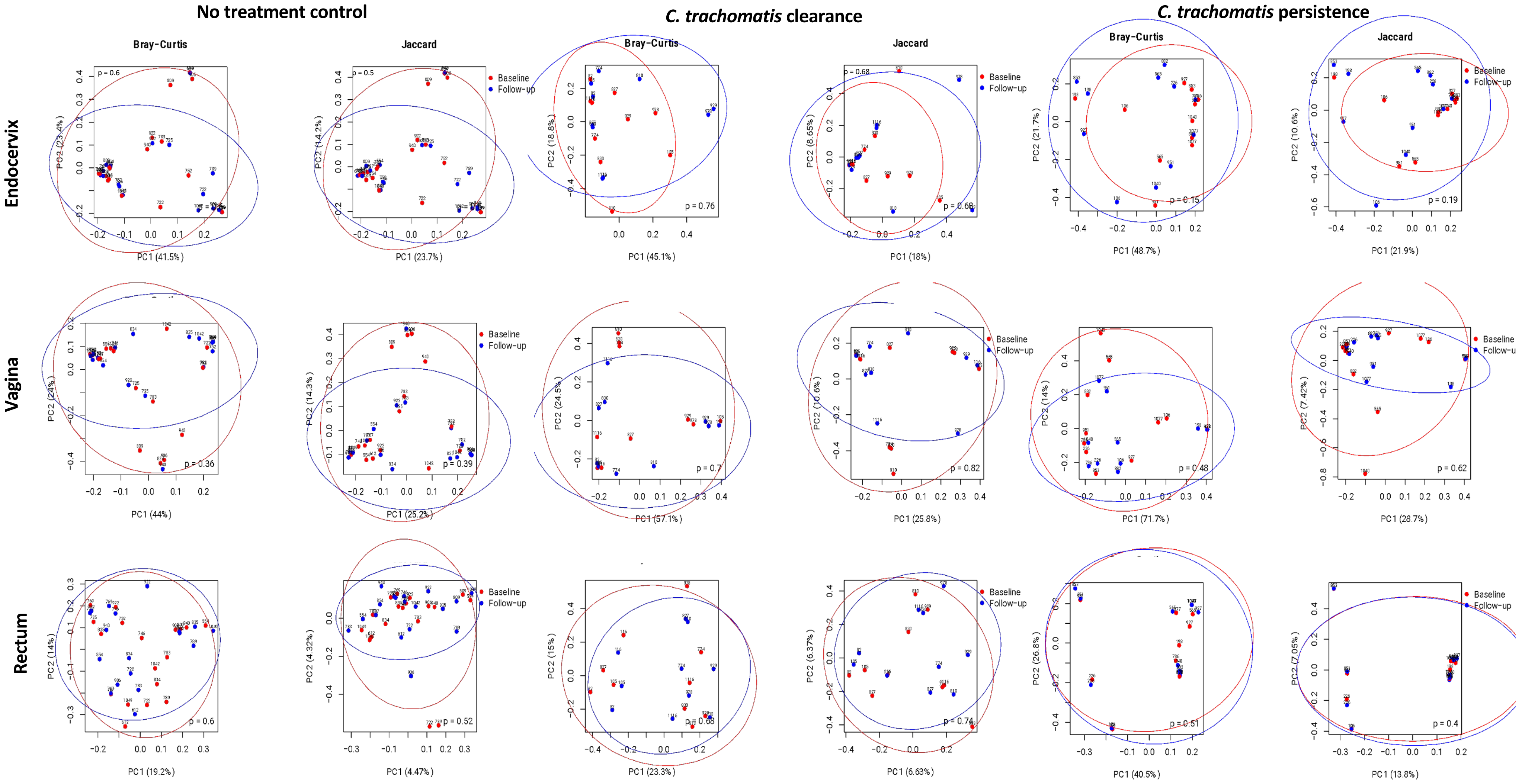

Supplementary Figure S3

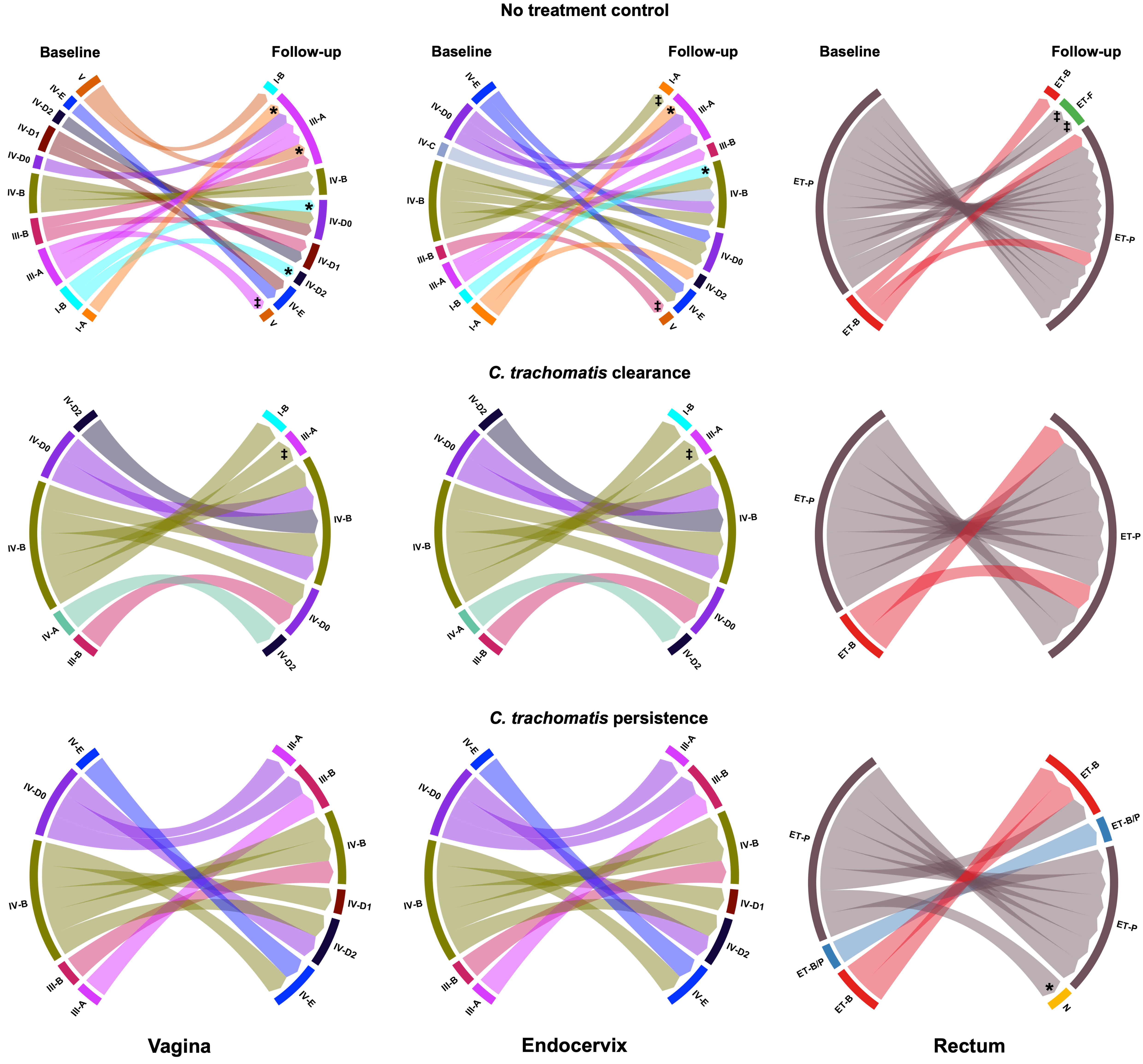

Supplementary Figure S4

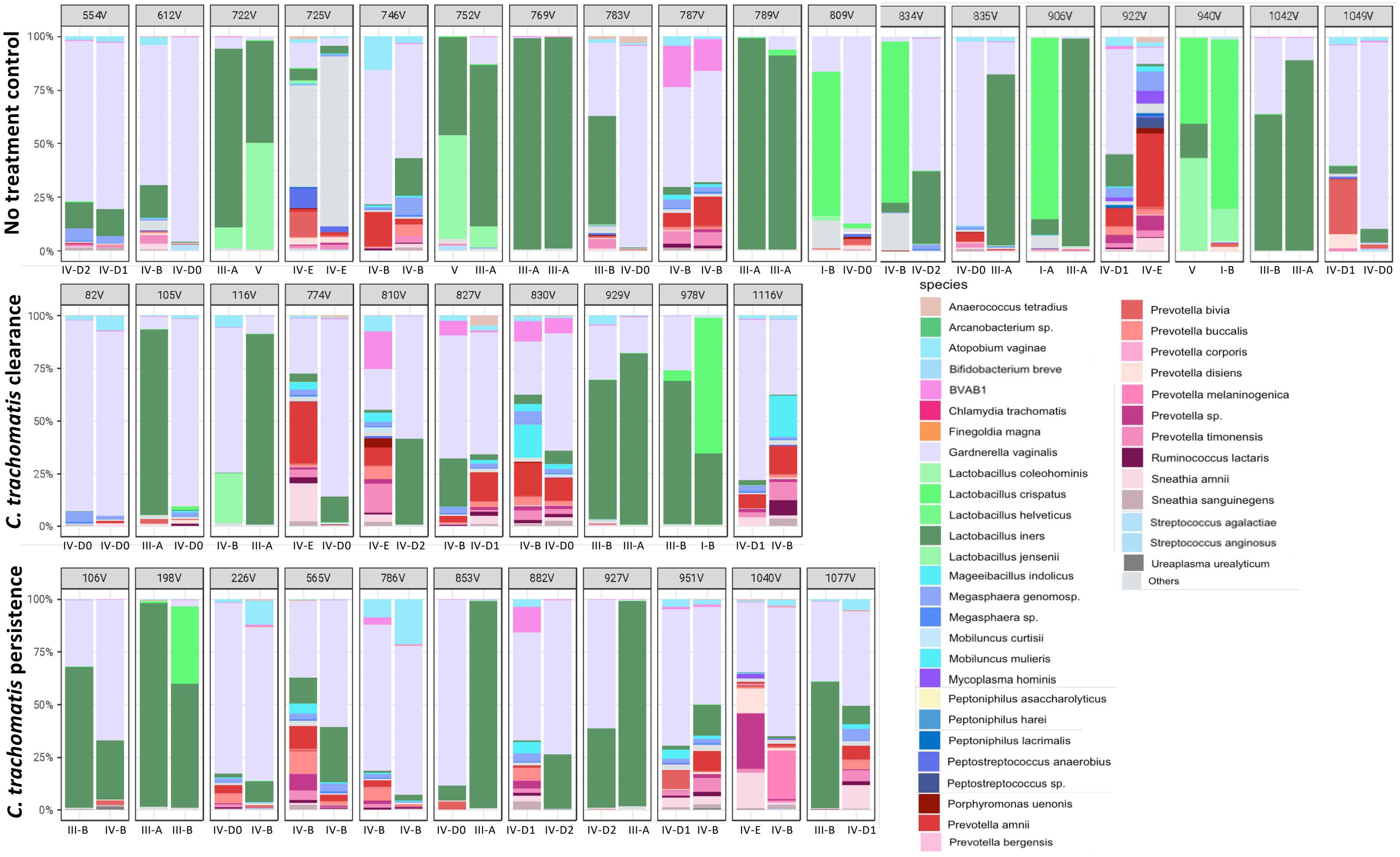

Supplementary Figure S5

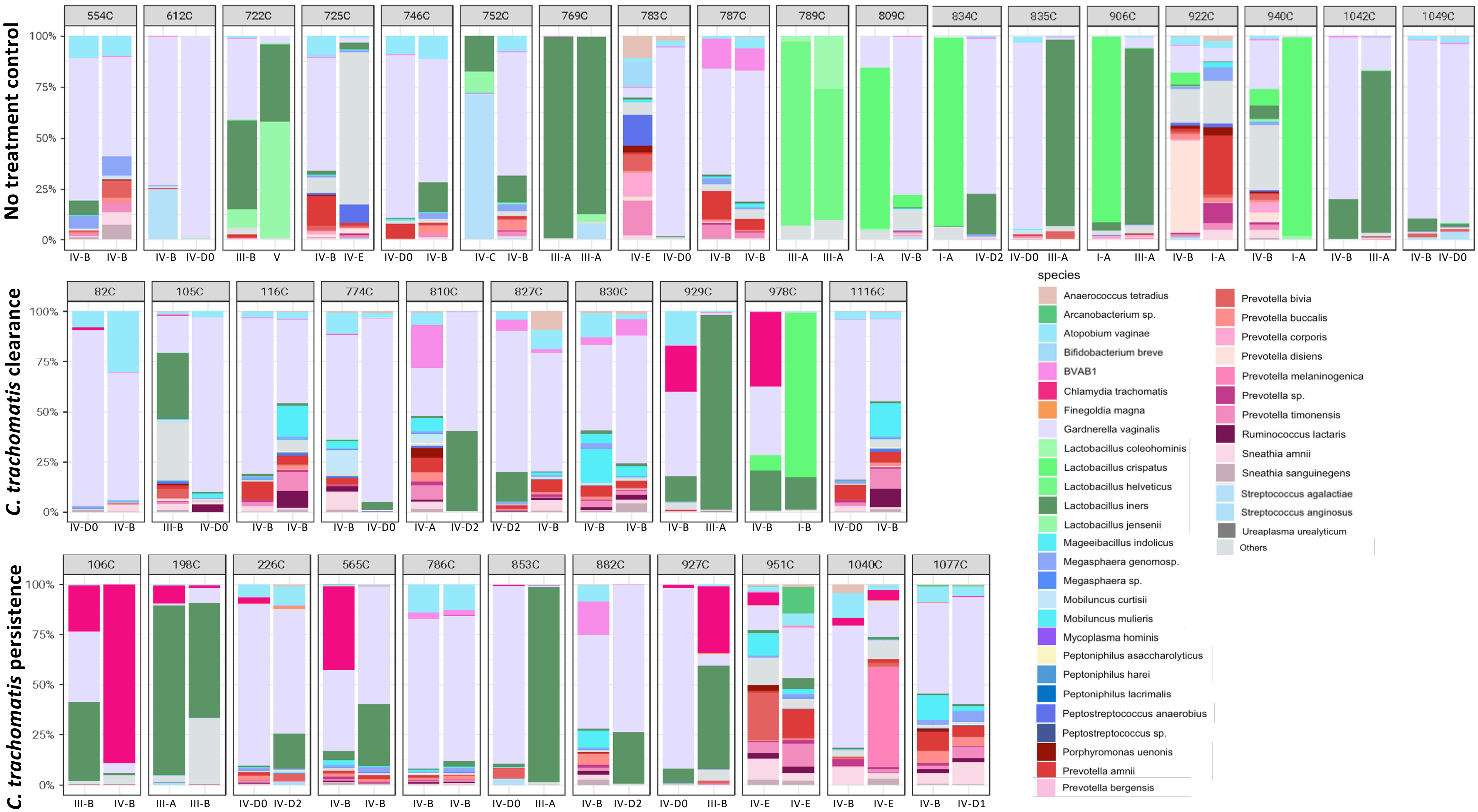

Supplementary Figure S6

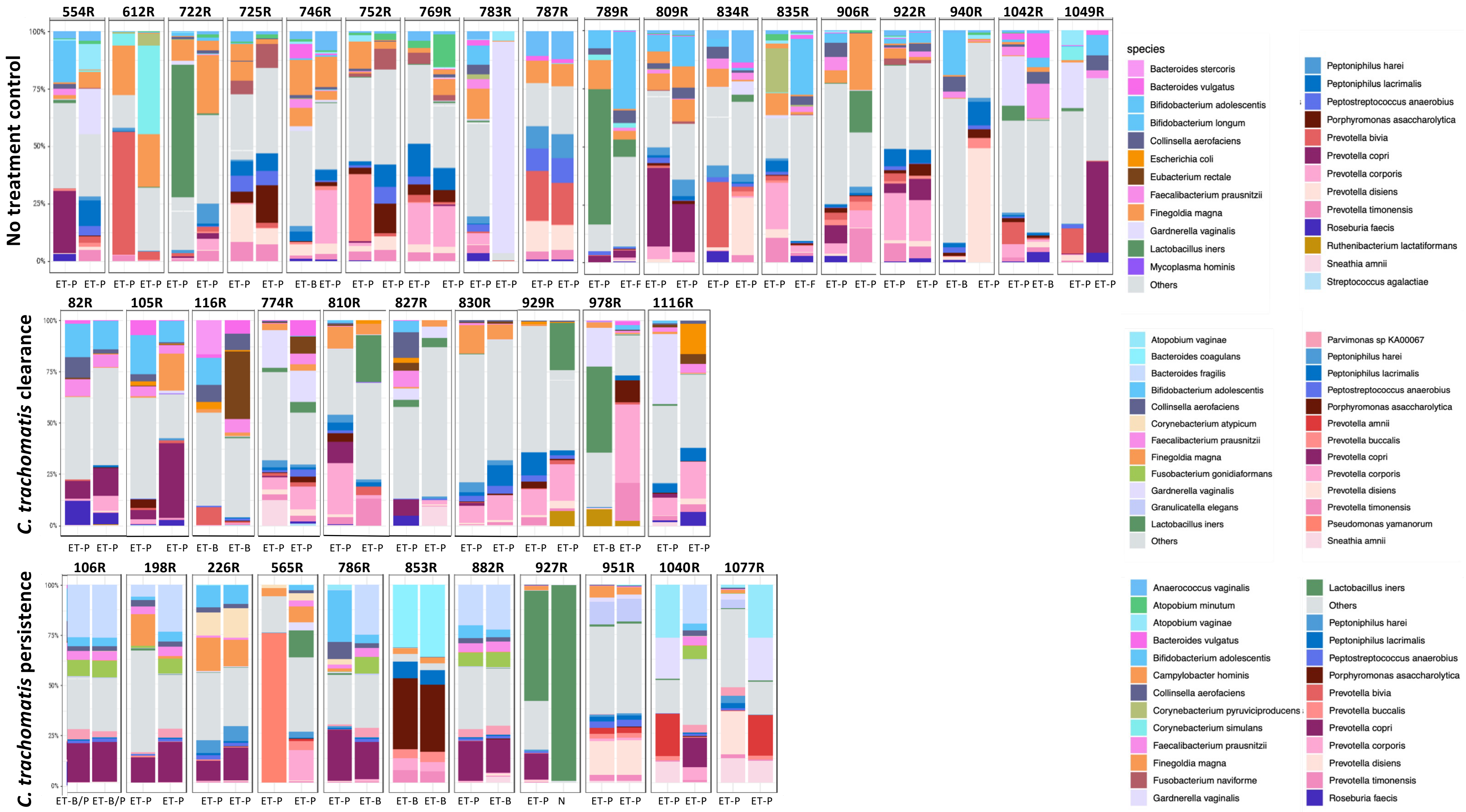

Supplementary Figure S7

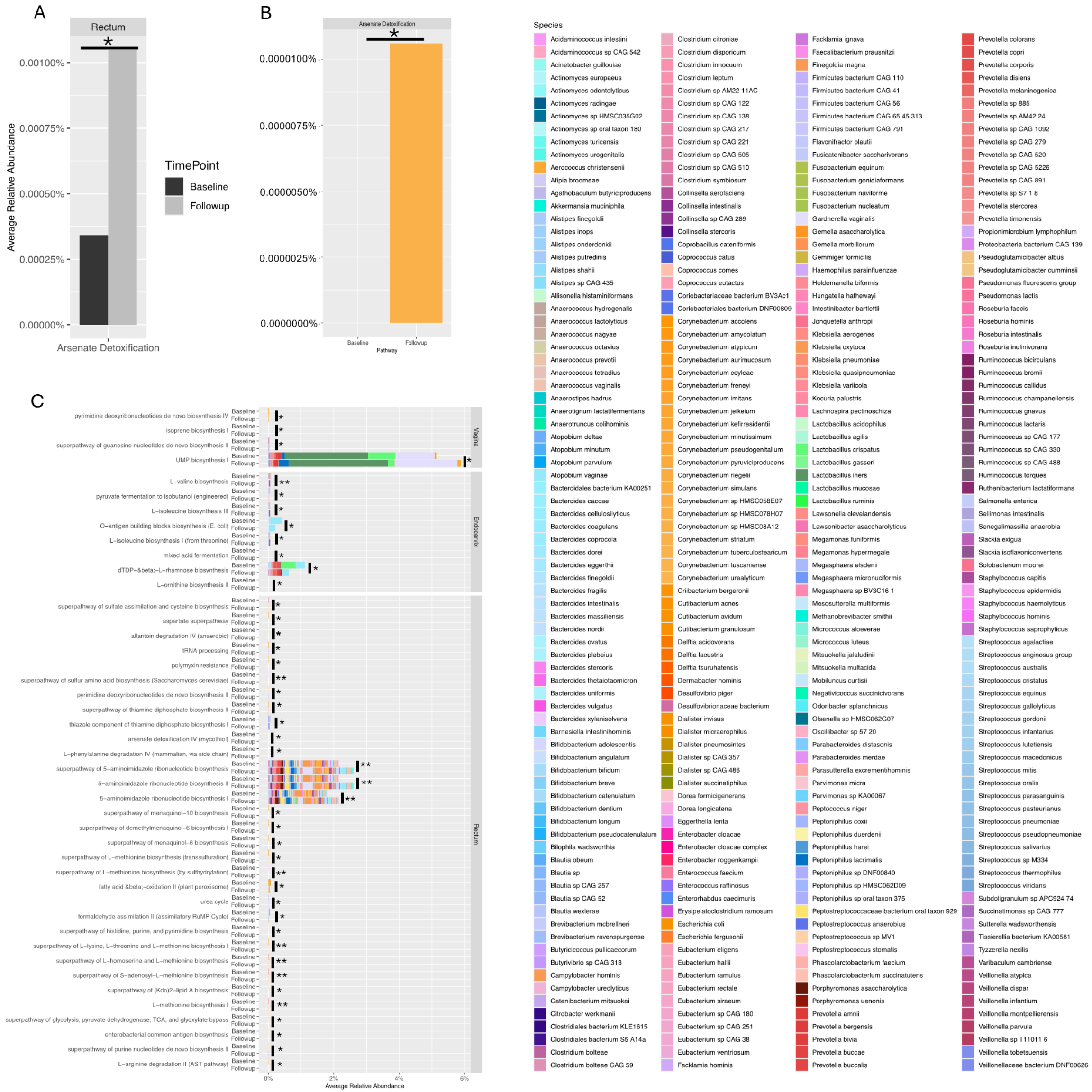

Figure S8

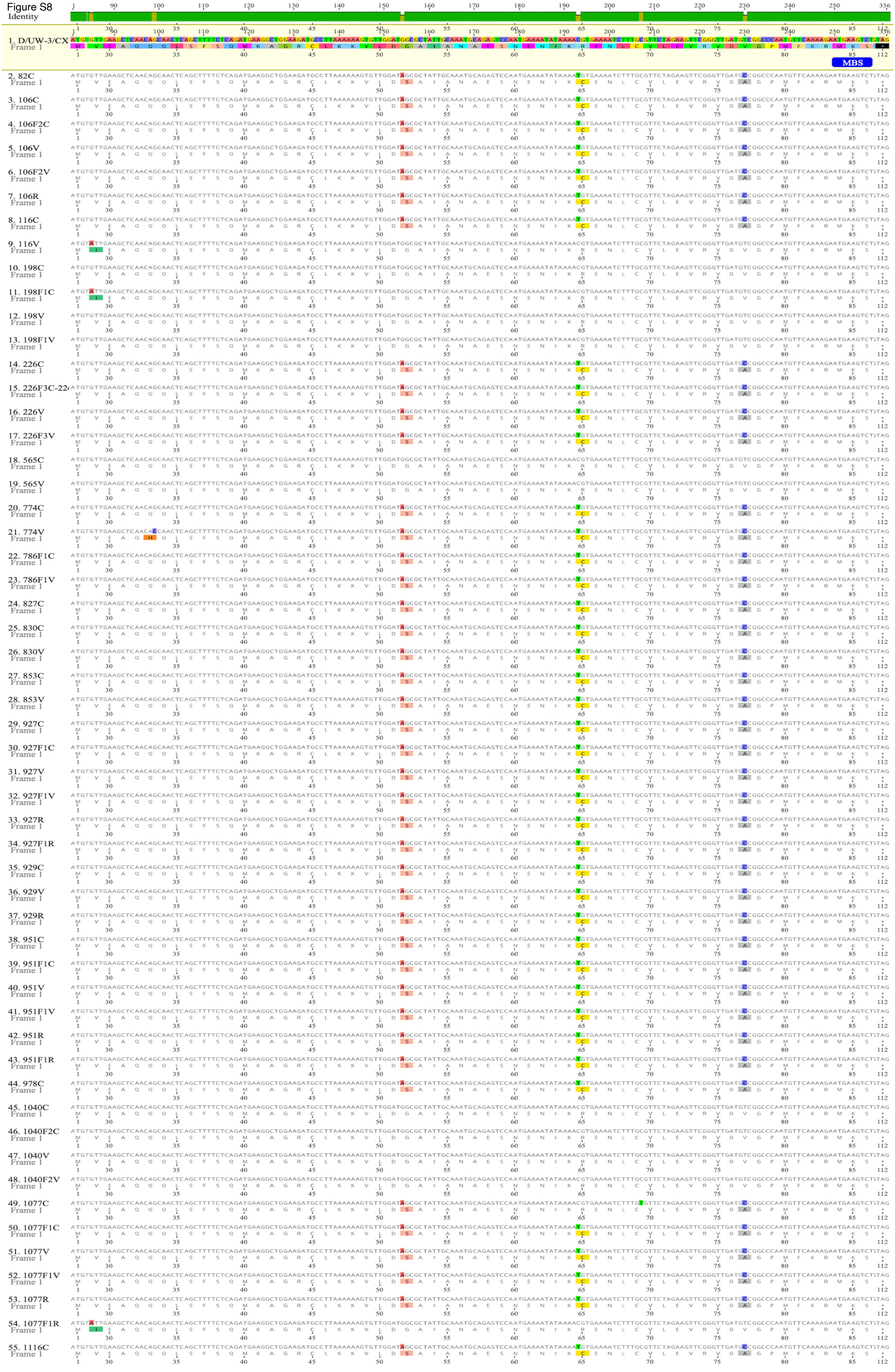
Identity
1. D/UW-3/CX
Frame 1
MBS
2. 82C
3. 106C
4. 106F2C
5. 106V
6. 106F2V
7. 106R
8. 116C
9. 116V
10. 198C
11. 198F1C
12. 198V
13. 198F1V
14. 226C
15. 226F3C-22
16. 226V
17. 226F3V
18. 565C
19. 565V
20. 774C
21. 774V
22. 786F1C
23. 786F1V
24. 827C
25. 830C
26. 830V
27. 853C
28. 853V
29. 927C
30. 927F1C
31. 927V
32. 927F1V
33. 927R
34. 927F1R
35. 929C
36. 929V
37. 929R
38. 951C
39. 951F1C
40. 951V
41. 951F1V
42. 951R
43. 951F1R
44. 978C
45. 1040C
46. 1040F2C
47. 1040V
48. 1040F2V
49. 1077C
50. 1077F1C
51. 1077V
52. 1077F1V
53. 1077R
54. 1077F1R
55. 1116C

Supplementary Figure S9

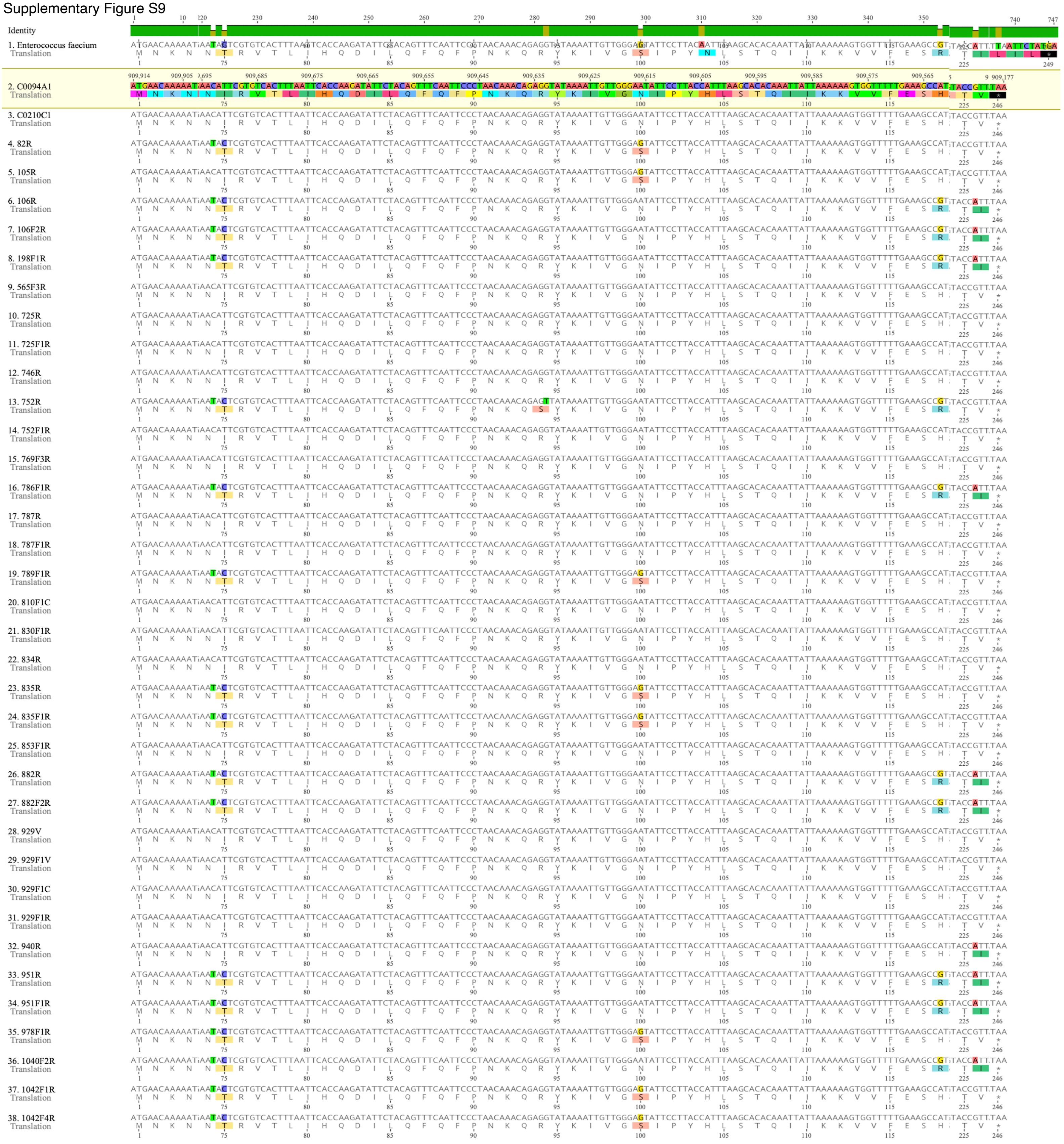

Figure S10

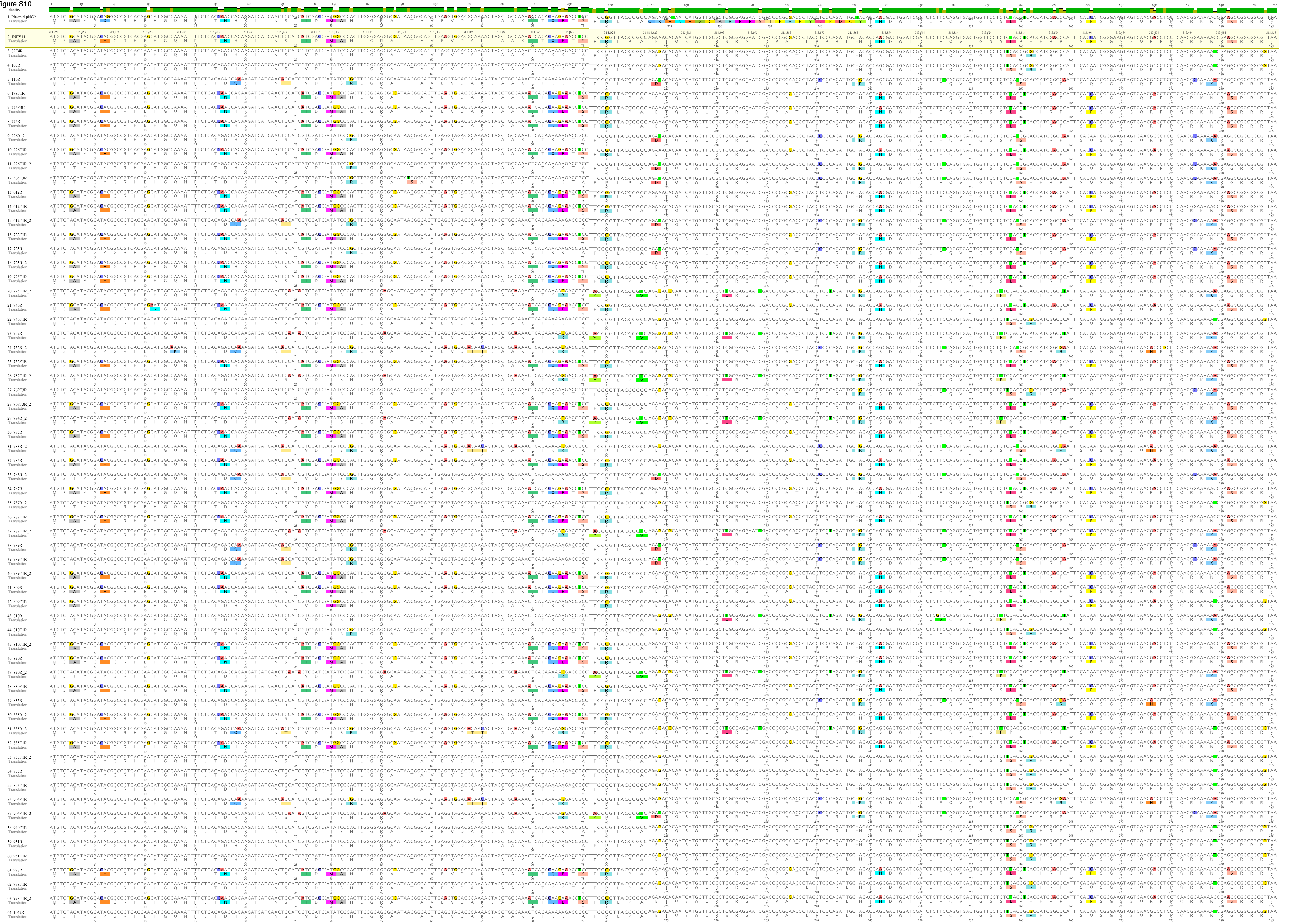

Supplement: Supplementary file 1 — Supplementary_Information. [file 41522_2025_858_MOESM1_ESM.pdf]
